# Tomato yield and water use efficiency change with various soil moisture and potassium levels during different growth stages

**DOI:** 10.1371/journal.pone.0213643

**Published:** 2019-03-27

**Authors:** Jie Liu, Tiantian Hu, Puyu Feng, Li Wang, Shuohuan Yang

**Affiliations:** 1 College of Water Resources and Architectural Engineering, Northwest A&F University, Yangling, Shaanxi, China; 2 Key Laboratory of Agricultural Soil and Water Engineering in Arid and Semiarid Areas of Ministry of Education, Northwest A&F University, Yangling, Shaanxi, China; Estacion Experimental del Zaidin, SPAIN

## Abstract

Faced with the scarcity of water resource and irrational fertilizer use, it is highly important to supply plants with water and fertilizer at desiderated stages to improve yield with high water use efficiency (WUE). A pot experiment was conducted to investigate the effects of growth stage-specific water deficiency and potassium (K) fertilization on tomato yield and WUE. The entire growing season of tomato was divided into 5 stages: vegetative growth stage (VG), flowering and fruit setting stage (FS), early fruit growth stage (FG), fruit development stage (FD) and fruit maturity stage (FM). Three soil moisture (W) and three K fertilization levels were set up. W levels included W1, W2 and W3, indicating that soil water was maintained at 60–70% field capacity, 70–80% field capacity, and 80–90% field capacity, respectively. K levels included K1, K2 and K3, indicating that 0 g K_2_O per kg soil, 0.46 g K_2_O per kg soil and 0.92 g K_2_O per kg soil was applied. All combinations of the three W and three K levels were solely imposed at each of the five growth stages, for other four stages, plants were watered to 80–90% field capacity without K fertilizer (W3K1). The permanent W3K1 over the entire growth stage was taken as control (CK). The results showed that W deficiency imposed at all stages significantly affected tomato yield (P<0.01), except for VG stage in which W deficiency did not cause yield loss. K fertilization level during FS or FM stage had a significant effect on yield (P<0.01). A significant interaction effect of W and K on yield was only observed during FM stage. For WUE, significant effect of W deficiency at FS, FD and FM stages were observed, and a significant effect of K levels at FS, FD and FM stages was observed. Specifically, K fertilization was necessary during specific growth stage of tomato (i.e. FS and FM). During FS stage, even if a sufficient water supply seems necessary, a deficit irrigation with K fertilization could be applied as K fertilization could alleviate the negative effect of soil water deficit, however, excess of K fertilization during FM stage should be avoided to maintain tomato yield and WUE.

## Introduction

In large areas of tomato (*Lycopersicon esculentum*) cultivation, rainfall could not meet crop water needs, and surface irrigation is mainly adopted by farmers in practice, which may lead to water waste [1]. Meanwhile, too much fertilizer applied in pursuit of high yield resulted in lower quality [2], soil salinization and groundwater contamination [3, 4]. Therefore, a better understanding of plant periodical response to soil moisture and fertilizer is highly important to water and fertilizer management in practice. To cope with water scarcity, which is the primary constraint for high crop yields in many arid areas, deficit irrigation (DI) has been widely adopted [5, 6]. DI allows the crop to experience a certain extent of water stress either by applying less irrigation during the entire crop cycle or by withdrawing irrigation at certain stages, without compromising yield too much. DI is demonstrated to increase water productivity and optimize water use efficiency (WUE) [7–9].

Crops are drought-sensitive at certain growth stages [10, 11], whereas they are drought-tolerant at other phenological stages [12–14]. In previous works, water stress imposed during fruit growth and maturity stages reduced tomato marketable yield [15], and the most sensitive period was the fruit maturity stage [14,16]. However, some researchers insist the most sensitive stage to water is the flowering and fruit setting stage [17, 18]. Although DI has been extensively investigated for many crops, the effect of DI applied at specific stages remains largely unknow.

Potassium (K) is one of the cationic minerals in most demand by tomato. K participates in plant photosynthesis, enzyme activation, protein synthesis and other biochemical and physiological processes [19, 20]. A deficiency of K causes direct disorder to these activities, which leads to a decrease in the growth rate of plant, accelerates leaf senescence and even leads to plant permanent wilting [21]. Additionally, K increases plant resistance to stress factors, such as drought, alkalinity and salinity [22–24]. K has a significant effect on the yield and its components of tomato [25, 26]. With K applied during the flowering stage, tomato yield and quality improved significantly [27, 28], and when applied during the reproductive stage, positive effects of K on vegetable dry biomass are observed [29]. However, increasing K application in field trials or nutrient solutions does not always improve tomato yield [30–32], but can decrease the economic benefit because excess K supply can reduce the mobility of calcium (Ca) thereby induced the occurrence of blossom-end rot [33].

Both water and K fertilizer are critical resources in agricultural production. Many studies have been conducted on the effect of irrigation [18, 34, 35] or K fertilizer [27, 36, 37] on tomato yield and WUE. The combined effect of these factors has also been investigated on soybean, beet, potato, wheat, barley and maize [38, 39]. Nevertheless, few literature reports concern combined effect of irrigation and K fertilizer on tomato, much less referring to different growth stages.

We hypothesized the response of tomato to water deficiency and K fertilization largely depended on the phenological stage. The objective of this study was 1) to assess at which growth stage water and K fertilizer levels are critical, and 2) to investigate the combined effect of soil moisture and K fertilizer and applied timing on tomato yield and WUE.

## Materials and methods

### Site description and materials

The pot experiment was conducted under a rain shelter at the Key Laboratory of Agricultural Soil and Water Engineering in Arid and Semiarid Areas of Ministry of Education, Northwest A&F University, Yangling, Shaanxi Province, China (34°20″ N, 108°04″ E and altitude of 521 m).

The soil used in the experiment was taken from the 0–20 cm layer in a local field and was classified as silty clay loam soil. The gravimetric field capacity (*θ*_*f*_) was 27%, and the content of organic matter, nitrate nitrogen, ammonium nitrogen and rapidly available K was 2.46 g·kg^-1^, 7.25 mg·kg^-1^, 3.93 mg·kg^-1^ and 104.9 mg·kg^-1^, respectively. The pots used in the experiment were 30 cm in height, 25 cm in bottom diameter and 30 cm in top diameter. Each pot was evenly filled with 20 kg of air-dried soil sieved through 5 mm diameter mesh, with a soil bulk density of 1.3 g·cm^-3^. Two polyvinyl chloride (PVC) tubes (2.5 cm in diameter, 30 cm in length) with 32 holes arrayed in four rows, intertwined by gauze (1 mm aperture) in case of soil clogging, were installed vertically in each pot to irrigate homogeneously. In this study, calcium magnesium phosphate (12.0% P_2_O_5_) and urea (46.4% N) were used as base fertilizers at 0.2 g P_2_O_5_·kg^-1^ soil and 0.12 g N·kg^-1^ soil. Additionally, an equal amount of urea acted as top dressing with irrigation at the fruit development stage of the first and second cluster, on May 31 and June14, 2015, respectively. Three clusters of tomatoes were remained. Other management activities were the same as local practices.

### Experimental design

Tomatoes (*Lycopersicon*. *esculentum* Mill. Jinpeng 10, local cultivar) were sown on March 12, and seedlings were separately transplanted to the pots on April 27, 2015, when 4–5 completely developed leaves appeared. 2 cm thickness of vermiculite was homogeneously spread on the soil surface to reduce evaporation from the pot. Each pot was irrigated to the field capacity immediately after transplantation. The tomato growth period was divided into five stages based on the first cluster of fruits, namely vegetative growth stage (VG, from transplanting to flowering, 5/5-11/5), flowering and fruit setting stage (FS, from flowering to fruit setting, 12/5-22/5), early fruit growth stage (FG, from fruit setting to fruit with 4cm diameter, 23/5-29/5), fruit development stage (FD, from fruit with 4cm diameter to fruit turning white, 30/5-20/6) and fruit maturity stage (FM, fruit turning from white to red, 21/6-25/7). Three water deficiency (W) and K fertilization levels were set up. W levels included W1, W2 and W3, indicating soil water was maintained at 60–70% field capacity, 70–80% field capacity and 80–90% field capacity, respectively. K fertilization levels included K1, K2 and K3, indicating 0 g K_2_O per kg soil, 0.46 g K_2_O per kg soil and 0.92 g K_2_O per kg soil was applied, respectively. All combinations of the three W and three K fertilization levels were solely imposed at each of the five growth stages, for other four stages, plants were well watered to 80–90% field capacity without fertilizer (W3K1). The permanent W3K1 over the entire growth stage was taken as the control (CK). Thus, the total treatments were 3×3×5–4 = 41.

Each treatment was replicated three times. Each pot was weighed daily during the experimental period to measure and control the soil water condition. K fertilizer was dissolved in water and applied with the irrigation water through the PVC tubes according to the above treatments.

### Measurements

Tomato fruits were picked and weighed when individual fruits reached maturity, and total yield (kg·plant^-1^) was calculated as the sum of the weights of all fruits for each plant.

WUE (kg·m^-3^) was calculated as the ratio of tomato yield and water consumption during the entire growth stage. We use this measurement as a proxy to tomato WUE in order to express water productivity of tomato. Seasonal water consumption was calculated as the sum of irrigation and the changes in soil water content between transplanting and harvesting (listed in S1 Table).

### Statistical analyses

The SPSS Statistics 21 software package (Version 21.0; IBM SPSS, Armonk, NY, USA) was used for analysis of variance (ANOVA). The significance of the effects of W, K fertilizer rates and their interaction was statistically evaluated at P<0.05 and P<0.01 significance levels. Duncan’s multiple range test was used for any significant differences among treatments [6]. Single effect analysis was performed when the interaction was significant. Graphs were plotted using Microsoft Excel 2016 (Microsoft Corporation, USA).

## Results

### General effects of timing and level of applied water and K fertilizer on yield and WUE

#### Tomato yield

Table 1 showed that as a whole, water and K fertilizer supplied stage had significant effect on tomato yield (P<0.001). Tomato yield was lowest when treatment was applied during FM stage, which was 15.77%, 14.36% and 14.51% lower than that applied during VG, FS and FG stages, respectively. Soil moisture level had significant effect on tomato yield (P<0.001). Plants grown under W1 possessed the lowest yield, which was 15.69% and 23.23% lower than W2 and W3, respectively. The effect of K fertilizer rate on yield was not significant (P>0.05). Stage × W level and stage × K fertilizer rate interactions were both significant (P<0.01), meaning the effect of W or K on tomato yield was closely related to the supply period. Generally, W level × K fertilizer rate, stage × W level × K fertilizer rate interactions had no significant effect on tomato yield (P>0.05).

**Table 1:** Output of three-way analysis of variance (ANOVA) and mean value of tomato yield and WUE as affected by the treatment stage, W level and K fertilizer rate.

Factors	Yield (kg plant^-1^)	WUE (kg m^-3^)
Treatment stage	***	***
VG	0.96a	24.33a
FS	0.94a	23.64a
FG	0.95a	23.78a
FD	0.90ab	23.16a
FM	0.81b	20.28b
W level	***	***
W1	0.78c	21.37b
W2	0.93b	23.39a
W3	1.02a	24.23a
K fertilizer rate	ns	**
K1	0.90	22.03b
K2	0.95	24.04a
K3	0.89	22.95ab
Stage×W level	***	***
Stage× K fertilizer rate	**	**
W level×K fertilizer rate	ns	ns
Stage× W level ×K fertilizer rate	ns	ns

*Note*: The table reported the significance results of the three-way ANOVA on tomato yield and WUE and each mean value of the main factors (VG, FS, FG, FD and FD; W1, W2 and W3; K1, K2 and K3) of tomato plants as affected by treatment stage, soil moisture level (W) and K fertilizer rate (K).

*, ** and *** indicate significance levels at P<0.05, P<0.01, and P<0.001, respectively; ns denotes no significance.

Different letters at sampling data under W, K and Stage treatments indicate significant difference between treatment according to Duncan multiple range test at P<0.05.

#### WUE

Water and K fertilizer supplied stage significantly affected tomato WUE (P<0.001, Table 1). Plants with W and K fertilizer controlled during FM stage had minimum WUE, 16.64%, 14.20%, 14.73% and 12.42% lower than those controlled during VG, FS, FG and FD stages, respectively. The effect of W level on tomato WUE was highly significant (P<0.001, Table 1). Plants grown under W1 had decreased WUE, 8.63% and 11.80% lower than those grown under W2 and W3, respectively. The effect of K fertilizer rate on tomato WUE was significant (P = 0.01, Table 1). Tomato WUE increased by 9.14% at K2 compared to K1. Stage × W level and stage×K fertilizer rate interaction were both significant (P<0.01, Table 1), meaning the effect of W or K on tomato WUE depended on the supply period. Globally, the effects of W level × K fertilizer rate, stage × W level × K fertilizer interactions on tomato WUE was not significant (Table 1).

### Effects of W level and K application rates at different growth stages on tomato yield

#### Vegetative growth (VG) stage

**Fig 1:**
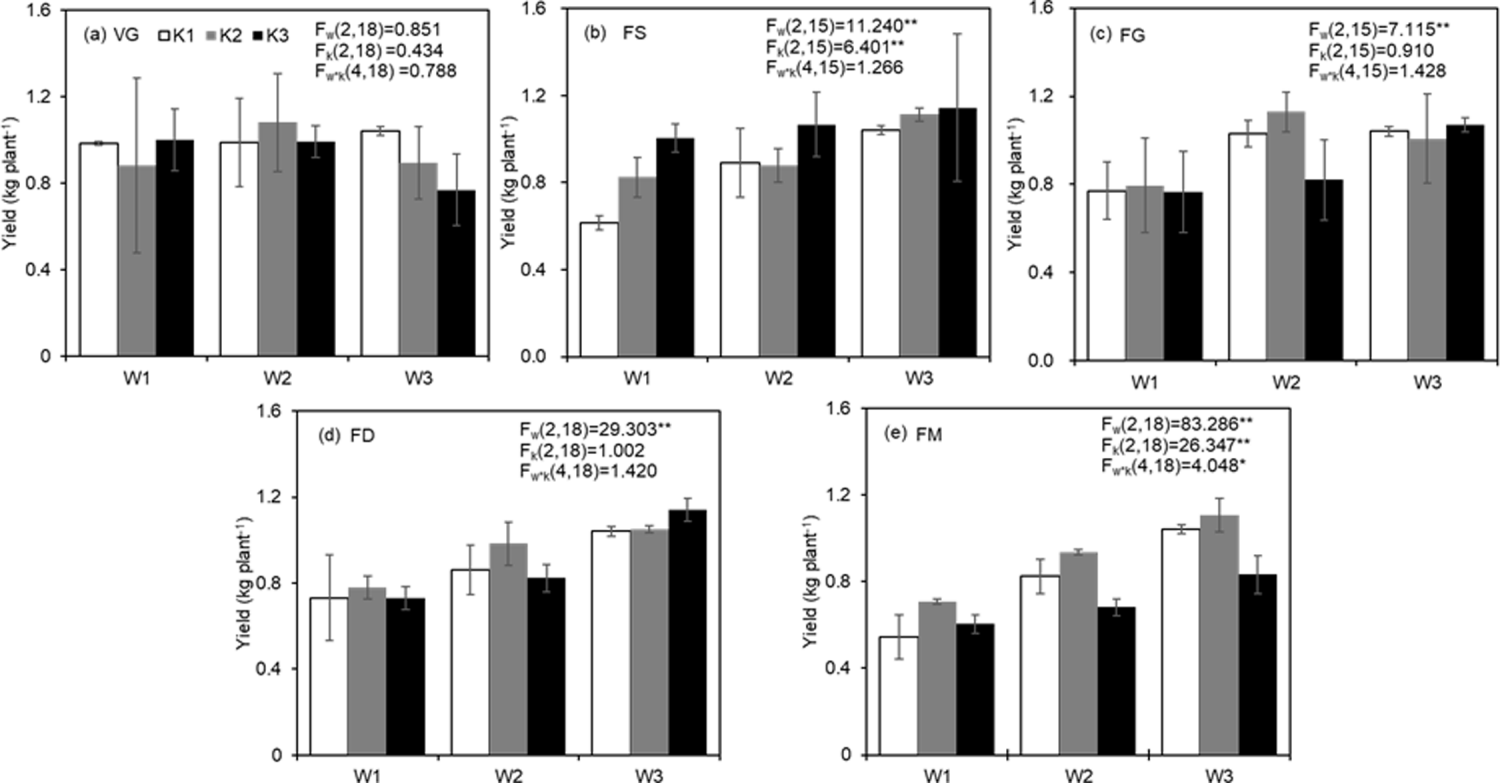
**Tomato yield as effected by soil moisture and K supply during vegetative growth (VG, a) stage, flower and fruit setting (FS, b) stage, fruit early growth (FG, c) stage, fruit development (FD, d) stage and fruit maturity (FM, e) stage.** W1, W2 and W3 denote three soil moisture levels, i.e., 60–70% *θ*_*f*_, 70–80% *θ*_*f*_ and 80–90% *θ*_*f*_, respectively. K1, K2 and K3 denote three K application rates, i.e., 0 g K_2_O·kg^-1^ soil, 0.46 g K_2_O·kg^-1^ soil and 0.92 g K_2_O·kg^-1^ soil, respectively. Error bars indicate standard error of the mean (n = 3 or 2). F_w_, F_k_ and F_w×k_ are the F values of the variance analysis for soil moisture, K rate and their interaction effect, respectively. The symbol of * and ** indicate significant effect at 0.05 and 0.01 level, respectively.

At the VG stage, the effect of W and K fertilizer on fresh yield was not significant (Fig 1A). Tomato yield varied from 0.65 to 1.08 kg·plant^-1^ without significant differences between CK and treatments. Nonetheless, yield tended to decrease with the increase in K level when W was maintained at 80–90%*θ*_*f*_, and the decrease was not significant.

#### Fruit setting (FS) stage

At the FS stage, both W and K fertilizer had a significant effect on tomato yield (P≤0.01), but their interaction was not significant (P>0.05, Fig 1B).

When soil water content was at W1, with the increasing application of K, tomato yield increased gradually, and compared with K1, K3 increased tomato yield by 63.29% (Fig 1B). However, no significant difference in the yield occurred among varied K-rate treatments when soil moisture was W2 or W3 (Fig 1B), indicating that K fertilizer at this stage could help to improve tomato yield under a water stress condition but not under situations of adequate soil moisture.

The result was similar for the effect of W on yield during the FS stage (Fig 1B). Compared with CK, W1K1 and W2K1 decreased yield by 40.88% and 14.31%, respectively. For the K fertilizer rate of K2, compared with W1, W3 increased tomato yield by 35.02%. For K3 no significant difference in yield occurred among different W levels (Fig 1B).

#### Early fruit growth (FG) stage

The influence of W at the FG stage on tomato yield was significant (P<0.01). Compared with W1, tomato yield increased by 27.23% and 33.86% at W2 and W3, respectively. K fertilizer and the interaction were not significant (P>0.05, Fig 1C).

During the FG stage, there was no significant difference in yield obtained by CK, W1K1 and W2K1. (Fig 1C). In the condition of K2, W2 significantly increased the yield (by 41.83%) compared to W1. Similarly, when the K fertilizer rate was K3, tomato yield acquired at W3 was 39.40% higher than that at W1.

No significant difference in tomato yield caused by K fertilizer rate was observed when soil moisture was W1 or W3. When the soil moisture was W2, yield tended to increase a little and then decreased sharply by 27.32% with increasing K application, and tomato yield reached the maximum (1.13 kg·plant^-1^) under the W2K2 treatment, indicating that moderate soil moisture and K application helped yield formation.

#### Fruit development (FD) stage

W at the FD stage had a highly significant influence on tomato yield (P<0.01, Fig 1D), which increased significantly with increasing W independent of the K fertilizer level (Fig 1D). Furthermore, compared with the tomato yield obtained at the soil moisture of W1, yield harvested at W3 increased by 46.00%, 35.22%, and 56.30% at the three different K rates, revealing that sufficient irrigation at the FD stage was vital to yield formation.

Neither the K fertilizer amount nor the interaction with W had a significant effect on yield (P>0.05, Fig 1D). Regardless of the soil moisture status, no difference in yield was detected among the treatments with varying K rates (Fig 1D).

#### Fruit maturity (FM) stage

At the FM stage, the main effects of both W and K application rate were highly significant (P<0.01), and their interaction was significant (P<0.05, Fig 1E).

Based on the significant interaction, single effect analysis was performed to explore the influence of different levels of W or K fertilizer on tomato yield (Table 2). At the FM stage, a significant difference in tomato yield occurred among K fertilizer treatments regardless of W status (P<0.05), and the difference was highly significant when soil moisture was W2 or W3 (P<0.01). When soil moisture was at W1, compared with the K1 rate, K2 increased tomato yield by 29.78% remarkably, whereas no difference in yield was observed between W1K3 and W1K1 treatments (Fig 1E). When the soil moisture was at W2, compared with the K-absence treatment, K2 increased tomato yield by 13.71%, whereas K3 decreased yield by 17.34%. For W3, no significant difference in yield was observed between CK and W3K2 treatment; however, compared with CK, the yield obtained by W3K3 was reduced by 24.82%. Single effect analysis also indicated that the difference in yield among W treatments was highly significant (P<0.01). Regardless of the K rate, tomato yield increased significantly with the increase in soil moisture (Fig 1E), indicating that sufficient water supply was conducive to tomato yield formation.

**Table 2:** Single effect of soil moisture level or K fertilizer rate during the fruit maturity stage on tomato yield.

Factor	Sum of squares	Degree of freedom	Mean square	F-value	P-value
K vs. W1	0.041	2	0.020	5.334	0.015*
K vs. W2	0.098	2	0.049	12.907	0.000**
K vs. W3	0.123	2	0.062	16.204	0.000**
W vs. K1	0.372	2	0.186	48.937	0.000**
W vs. K2	0.242	2	0.121	31.816	0.000**
W vs. K3	0.081	2	0.040	10.630	0.001**

*Note*: W1, W2 and W3 denote three levels of soil moisture, i.e., 60–70%, 70–80% and 80–90% *θ*_*f*_, respectively. K1, K2 and K3 denote three rates of K fertilizer, i.e., 0, 0.46 and 0.92 g K_2_O·kg^-1^ soil, respectively.

* indicates a significant effect (P<0.05), and

** indicates a highly significant effect (P<0.01).

Fig 1E also shows that the maximum yield (1.107 kg·plant^-1^) was obtained when soil moisture was W3 and the dose of K fertilizer was K2, with yield increasing by 103.49% compared with the minimum yield (0.544 kg·plant^-1^) obtained by W1K1.

### Effects of W and K application rates at various growth stages on WUE of tomato

The interaction of stage×W and stage×K rate were both significant (P<0.01, Table 1).

**Fig 2:**
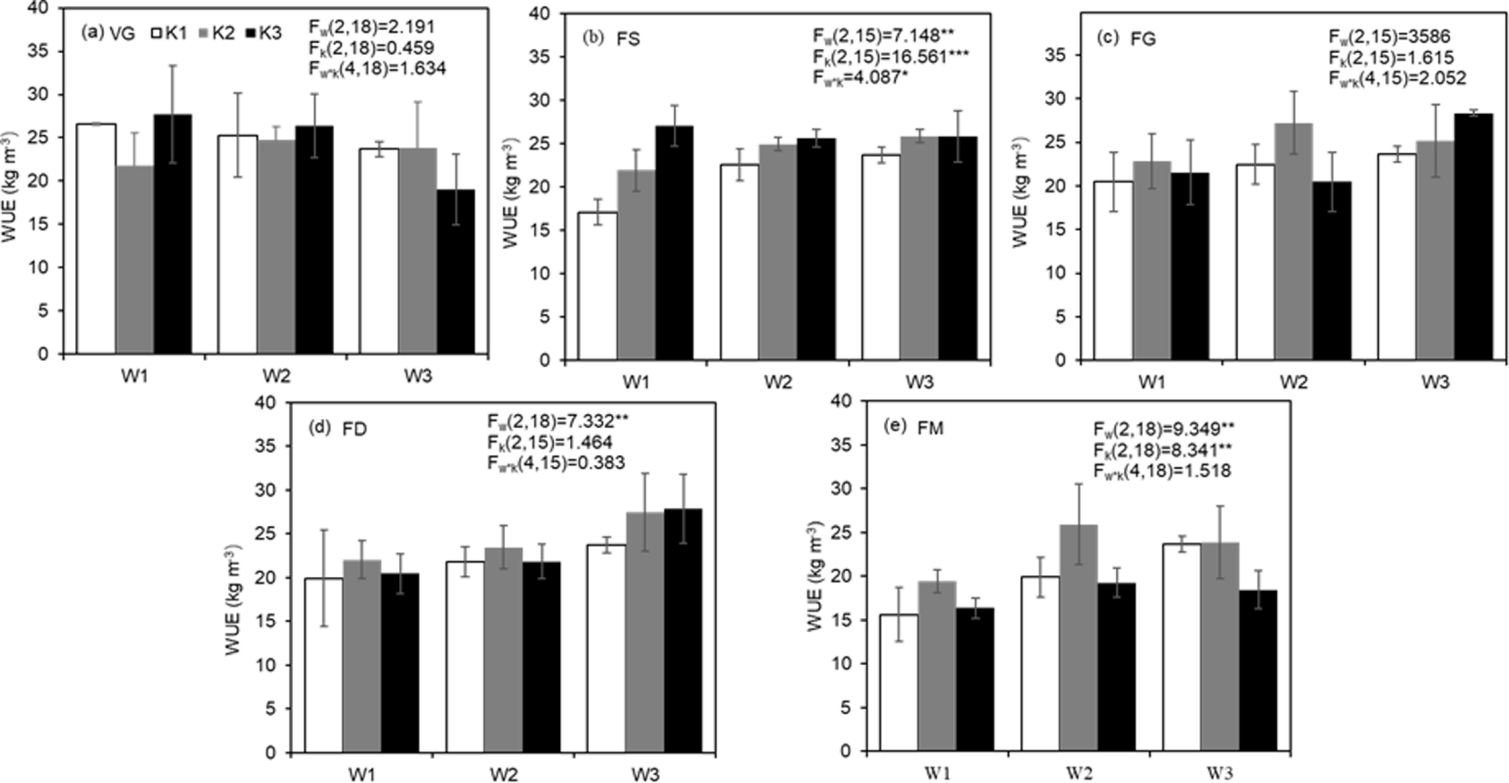
**Tomato water use efficiency (WUE) as effected by soil moisture and K supply during vegetative growth (VG, a) stage, flower and fruit setting (FS, b) stage, fruit early growth (FG, c) stage, fruit development (FD, d) stage and fruit maturity (FM, e) stage.** W1, W2 and W3 denote three soil moisture levels, i.e., 60–70% *θ*_*f*_, 70–80% *θ*_*f*_ and 80–90% *θ*_*f*_, respectively. K1, K2 and K3 denote three K application rates, i.e., 0 g K_2_O·kg^-1^ soil, 0.46 g K_2_O·kg^-1^ soil and 0.92 g K_2_O·kg^-1^ soil, respectively. Error bars indicate standard error of the mean (n = 3 or 2). F_w_, F_k_ and F_w×k_ are the F values of the variance analysis for soil moisture, K rate and their interaction effect, respectively. The symbol of * and ** indicate significant effect at 0.05 and 0.01 level, respectively.

During the VG or FG stage, W and K application rate had little effect on WUE of tomato, and the interactions were also not significant (P>0.05, Fig 2A and 2C).

During FS stage, the effect of W levels on tomato WUE was significant (P<0.01, Fig 2B). W3 plants possessed 13.78% higher WUE than W1 plants. The effect of K rates on WUE was also significant (P<0.01). Compared to K1, K2 and K3 improved WUE by 14.43% and 24.52%, respectively. The interaction of W and K application was significant (P<0.05). With soil water deficit at W1 level, in relation to K1, K2 and K3 enhanced WUE by 28.19% and 58.14%, respectively. With K level at K1 rate, compared with W1, W2 and W3 increased WUE by 32.04% and 38.52%, respectively.

During the FD stage, W had a significant effect on tomato WUE (P<0.01, Fig 2D). Plants grown with soil moisture at W3 possessed 26.49% and 17.68% higher WUE than those grown at W1 and W2, respectively. The effects of K fertilizer rate and the interaction with W were not significant (P>0.05, Fig 2D). Tomato WUE was highest in treatments W3K2 (27.44 kg·m^-3^) and W3K3 (27.82 kg·m^-3^) and lowest in those of W1K1 (19.91 kg·m^-3^) and W1K3 (20.46 kg·m^-3^), suggesting that increasing soil moisture at the fruit development stage was helpful for tomato WUE.

During the FM stage, both W and K fertilizer rate affected WUE significantly (P<0.01), whereas their interaction was not significant (P>0.05, Fig 2E). At K1 level, WUE increased from 15.61 kg·m^-3^ to 23.67 kg·m^-3^ with increasing soil moisture. The maximum value was obtained for W2K2 (25.93 kg·m^-3^), which was 30.27% and 34.70% higher than that of W2K1 and W2K3, respectively (Fig 2E).

## Discussion

### Yield change by soil moisture varies with the controlled growth stage

Irrigation plays a vital role in tomato yield formation, and the effect varies from stage to stage. In this study, soil moisture (W) at the vegetative growth (VG) stage had little effect on tomato yield (Fig 1A). This result could be explained by that during this period, the temperature was relatively low and plants only had 5–6 leaves, resulting in low evapotranspiration. Thus, soil moisture of 60~70%*θ*_*f*_ was adequate to meet the demand of water for tomato plants [14], and water-saving strategies are recommended at this stage.

In this research, soil moisture at the flowering and fruit setting stage (FS), early fruit growth stage (FG) and fruit development (FD) or maturity stage (FM) had highly significant effects on tomato yield (Fig 1B–1E). This result was in consistent with that of Chen et al, who reported that excessive and insufficient soil moisture during FS stage had negative effects on tomato yield, while increasing soil moisture during the last three stages could significantly promote tomato yield [14]. Additionally, soil water deficit during flowering and/or yield formation stages sharply reduces the marketable yield of tomato [13, 35]. For non-K or moderate K treatments at the FS stage, low soil moisture (60–70% *θ*_*f*_) significantly decreased tomato yield (Fig 1B). This decrease could be explained by the fact that leaf stomatal conduction declines and stomatal resistance increases when soil moisture was 60–70% *θ*_*f*_, which inhibits the rate of photosynthesis [40]. The demand of tomato for water is high from the FS stage to the FM stage, particularly at the FD and maturity stage when water is a limiting factor for tomato plant growth. Soil water deficit during the fruit development and ripening stage restricts the movement of calcium from soil to fruit and results in blossom-end rot and yield loss [41]. In this study, with the decrease in soil moisture at the FD and FM stages, tomato yield decreased significantly (Fig 1D and 1E), which was consistent with the findings of Marouelli & Silva [42]. The decline in yield by DI at the FD and FM stages could be caused by inhibited cell multiplication and expansion and restricted translocation of assimilates from leaves to fruit [39]. Moreover, water deficit could influence the fruit setting and development of the second or third cluster of fruits resulting in reductions in fruit weight and fruit number (S2 Table), which is consistent with the results obtained by Nangare et al. [18]. Furthermore, sufficient water supply at these two stages is crucial to meet the demand of water for high-intensity evapotranspiration induced by high temperatures and flourishing leaf area, and vital for enlargement of fruit cells and metabolism of fruit nutritional substances; therefore, single fruit weight increased in treatments with sufficient water supply compared with those exposed under soil moisture deficit (S2 Table).

### Yield change by K fertilizer rate varies with the controlled growth stage

In this study, tomato yield had highly significant response to K application at the FS stage or the fruit maturity stage, whereas little response was observed at the VG, FG or FD stage (Fig 1A–1E). Han et al. also reported that potash applied at the flowering phase not only increases plant height and stem width but also increases yield, whereas potash applied at the fruit enlarging phase had no statistical effect on yield [28]. At the FS stage, when soil moisture was 60–70% *θ*_*f*_, yield tended to decline with decreased K application (Fig 1B). This decline could be explained by that K deficiency at FS stage decelerates the photosynthesis rate and translocation of assimilates from source (e.g., leaves) to sink (e.g., fruits) [43, 44], which contributed to the decrease in fruit weight (S2 Table). At FM stage, the medium K rate helped to obtain the greatest yield, while sufficient K fertilization appeared to decrease tomato yield (Fig 1E). Similar results were reported by previous studies [26, 33, 45]. This result might be related to that high K supply reduced the mobility of Ca and consequently, increased the occurrence of blossom-end rot [33]. Excess application of K fertilizer could also reduce boron (B) absorption, thus inducing tomato fruit microcracks and sacrifice quality [45]. Additionally, the benefit of K fertilization under soil moisture stress was only shown in FS stage. Thus, it’s beneficial to apply adequate K fertilizer during FS stage if water is limited, but not necessary during FM stage, and in this study for plants with three clusters, the K in the soil could meet plants demand under adequate soil moisture status.

### Interaction of W and K fertilizer on tomato yield

During the FM stage, the interaction of W and K fertilizer occurred (Fig 1E), indicating the ability of K to improve tomato yield was closely associated with soil moisture. During the FM stage of the first cluster, the second and third clusters of fruits were at the stage from FS to FD, which contributes much to the yield. Moreover, the temperature and evapotranspiration were high, leading to a high demand for water. Increase of soil moisture (i.e., W2 and W3) significantly improved yield, especially at K1 and K2 status (Fig 1E). As K is an important osmoticum in determining cell turgor and stomatal aperture for plants [46, 47], plant osmotic adjustment and photosynthesis are directly related to K nutrition status. K fertilizer can alleviate the negative effect of soil water deficit (Fig 1E) [48], while adequate K may lead to ion antagonism (i.e. K and magnesium), resulting an unbalanced nutrition status in soil, which in turn affect crop yield [49]. Additionally, overuse of K may reduce Ca mobility and induce the bottom-end rot [33]. In this study, medium K supply improved tomato yield in regardless of soil water status, this could be related to that medium K enhanced osmotic adjustment and sustained cell expansion, increasing drought resistance potential for plants [22, 50], and consequently, determined yield at the critical stages of yield formation [48].

During FS stage, when soil moisture was limited as W1, the increased K supply improved yield. When soil moisture was sufficient as W3, tomato yield was constant with increased K supply (Fig 1B). This indicated the interaction of W and K fertilizer occurred during this stage despite that it is not significant as a whole according to analysis of variance (Fig 1B). At FS stage plant was more sensitive to soil water deficit (F_w_ = 11.240) than K fertilizer (F_k_ = 6.401) (Fig 1B). Lower soil moisture may lead to the flower abortion and fewer fruits (S2 Table). Moreover, K uptake by plant might be decreased at low soil water condition because the diffusion of K ion to roots from soil was inhibited [51,52], and K deficiency may impair photosynthesis activities and decelerate tomato plant growth and result in reduction in assimilate translocation to fruits [48]. In low soil moisture condition, sufficient K fertilizer to plants might enhance resistance to drought stress [22, 50] and alleviate the negative effect of soil water deficit [53]. This finding needed further investigation.

### WUE change by W and K fertilizer varies with controlled growth stage

Management of watering and fertilization not only change plant transpiration by participating in the fundamental physiological process [54, 55], but also change the surface evaporation of soil where the plant stands by direct and indirect process [56]. Moreover, the water amount of plant transpiration and soil evaporation has close interaction with each other [57]. In order to select agronomic measures to use water resources efficiently, soil evaporation should also be concerned besides plant transpiration. In other words, water productivity in the whole system of plant and its soil should be focused on. So, in this paper, WUE was calculated as the ration of tomato yield and water consumption during the entire growth stage, using this measurement as a proxy to tomato WUE.

WUE reflects the efficient use of water in crop production. Compared with the entire growing season, WUE is more sensitive to water during some phenological growth stages [13]. In our study, tomato WUE was defined as the ratio between total yield and seasonal water consumption (soil evaporation included). As all pots were in the same air condition, evaporation could be relatively similar for each pot. However, different soil moisture might cause difference in evaporation, which was linearly related with yield production [58]. Sato et al. [59] and Adams et al. [60] reported that poor fruit set was observed at high temperatures (more than 26°C). In this study, temperature in the air was higher during the last two stages. For treatments conducted during FD and FM stage, the maximum values of tomato WUE were observed at 80–90% *θ*_*f*_ and 70–80% *θ*_*f*_, and the minimum values were obtained at 60–70 *θ*_*f*_, which were consistent with the results of Nuruddin et al. who found that tomato WUE declined with water stress during fruit growth and ripening stages [15]. This might be related to the higher temperature in the plant leaf with lower evaporation when soil moisture was 60–70 *θ*_*f*_. However, during FS or FG stage, low soil moisture did not decrease WUE, which might be ascribed to the compensation effect resulting from sufficient water supply in FD and FM stages [61]. Additionally, during VG stage, low soil moisture did not increase tomato WUE (Fig 2A), which is different from the expectation. The reason might be the difference among the three W levels (60–70% *θ*_*f*_, 70–80% *θ*_*f*_, 80–90% *θ*_*f*_) was not sufficiently large to significantly affect the total water consumption in this investigation (S1 Table). This should be investigated further.

The effect of K fertilizer during FM stage on tomato WUE depended on the soil moisture status, and WUE was highest at 70–80% *θ*_*f*_ with 0.46 g K_2_O·kg^-1^ soil (Fig 2E). For arid and semiarid areas with a climate similar to that in this experiment, saving water resources during the fruit growth and ripening stage is important, which generally experiences the longest time in tomato production. With consideration of K fertilizer, soil moisture of 70–80% *θ*_*f*_ is recommended.

## Conclusion

Our study shows that the effects of soil water deficit and potassium fertilizer on tomato yield and water use efficiency depended on tomato growth stages. Soil moisture and potassium fertilizer were generally not limited factors during vegetative growth stage, so deficit irrigation without K fertilizer at this stage could be recommended. Potassium fertilizer could alleviate the negative effect of water stress during flower and fruit setting stage. Sufficient water supply during fruit development and maturity stage was essential for tomato yield. During fruit maturity stage tomato yield was enhanced by moderate potassium fertilizer, while decreased by excess application, indicating moderate level of potassium fertilizer during fruit maturity stage was needed.

## Supporting information

S1 Table Water consumption during the entire growth period (m^3^·plant^-1^). (DOCX)

S2 Table Single fruit weight and fruit number per plant of the same combination of W and K as affected by the growth stages of its supply. (DOCX)
