# Supplementary material for: Tomato yield and water use efficiency change with various soil moisture and potassium levels during different growth stages
Source: PLoS One. 2019 Mar 27;14(3):e0213643. doi: 10.1371/journal.pone.0213643 (PMC6436690; doi:10.1371/journal.pone.0213643)
Supplement: S1 Table — (DOCX) [file pone.0213643.s001.docx]

S1 Table. Water consumption during the entire growth period (m^3^·plant^-1^)

| Combination of  W and K | Control Period of Water and K | | | | |
| --- | --- | --- | --- | --- | --- |
|  | T_VG_ | T_FS_ | T_FG_ | T_FD_ | T_FM_ |
| W1K1 | 0.0370 | 0.0363 | 0.0383 | 0.0368 | 0.0349 |
| W1K2 | 0.0393 | 0.0377 | 0.0355 | 0.0355 | 0.0365 |
| W1K3 | 0.0364 | 0.0374 | 0.0354 | 0.0358 | 0.0370 |
| W2K1 | 0.0391 | 0.0394 | 0.0439 | 0.0394 | 0.0414 |
| W2K2 | 0.0435 | 0.0367 | 0.0417 | 0.0421 | 0.0370 |
| W2K3 | 0.0378 | 0.0407 | 0.0399 | 0.0379 | 0.0355 |
| W3K2 | 0.0379 | 0.0431 | 0.0399 | 0.0390 | 0.0471 |
| W3K3 | 0.0405 | 0.0431 | 0.0377 | 0.0414 | 0.0451 |

*Note*: T_VG_, T_FS_, T_EG_, T_FD_ and T_FM_ are the periodical soil moisture and K control at the vegetative growth stage, flowering and fruit setting stage, early fruit growth stage, fruit development stage and fruit maturity stage, respectively. Water consumption for the control treatment W3K1 was 0.0440 m^3^ ∙ plant^-1^.
