# Supplementary material for: Tomato yield and water use efficiency change with various soil moisture and potassium levels during different growth stages
Source: PLoS One. 2019 Mar 27;14(3):e0213643. doi: 10.1371/journal.pone.0213643 (PMC6436690; doi:10.1371/journal.pone.0213643)
Supplement: S2 Table — (DOCX) [file pone.0213643.s002.docx]

S2 Table. Single fruit weight and fruit number per plant of the same combination of W and K as affected by the growth stages of its supply.

| Combination of W and K | Single fruit weight (g) | | | | |  | | Fruit number per plant | | | | |
| --- | --- | --- | --- | --- | --- | --- | --- | --- | --- | --- | --- | --- |
|  | T_VG_ | T_FS_ | T_FG_ | T_FD_ | T_FM_ |  | T_VG_ | | T_FS_ | T_FG_ | T_FD_ | T_FM_ |
| W1K1 | 110.29 | 74.55c | 90.37c | 80.60d | 67.59c |  | 9.00 | | 8.33ab | 8.50b | 9.00 | 8.00c |
| W1K2 | 99.52 | 103.22ab | 93.34bc | 84.26d | 106.39b |  | 8.67 | | 8.00b | 8.50b | 9.33 | 6.67d |
| W1K3 | 97.20 | 104.46ab | 92.70bc | 95.45bcd | 79.34c |  | 9.50 | | 9.67ab | 8.33b | 7.67 | 7.67cd |
| W2K1 | 115.80 | 95.11b | 97.99abc | 103.12abc | 99.13b |  | 8.67 | | 9.33ab | 10.50ab | 8.33 | 8.33bc |
| W2K2 | 112.42 | 97.63b | 94.06bc | 106.00abc | 112.73ab |  | 8.50 | | 9.00ab | 12.00a | 9.33 | 8.33bc |
| W2K3 | 99.15 | 112.10ab | 74.13d | 92.31cd | 102.96b |  | 10.00 | | 9.50ab | 11.00ab | 9.00 | 6.67d |
| W3K1 | 108.02 | 108.02ab | 108.02ab | 108.02abc | 108.02b |  | 9.67 | | 9.67ab | 9.67ab | 9.67 | 9.67a |
| W3K2 | 102.14 | 111.33ab | 112.99a | 109.13ab | 118.79ab |  | 9.00 | | 10.00a | 9.00ab | 9.67 | 9.33ab |
| W3K3 | 110.48 | 119.35a | 103.76abc | 118.14a | 108.02b |  | 7.00 | | 9.50ab | 10.33ab | 9.67 | 7.67abc |
| Source P Value of Significance Test | | | | | | | | | | | | |
| W | 0.644 | 0.002** | 0.000** | 0.000** | 0.000** |  | 0.751 | | 0.079 | 0.012* | 0.144 | 0.000** |
| K | 0.419 | 0.002** | 0.034* | 0.517 | 0.003** |  | 0.871 | | 0.471 | 0.901 | 0.423 | 0.013* |
| W$\times$K | 0.712 | 0.115 | 0.111 | 0.073 | 0.066 |  | 0.227 | | 0.385 | 0.671 | 0.483 | 0.032* |

*Notes*. T_VG_, T_FS_, T_FG_, T_FD_ and T_FM_ are the periodical water and K control during the vegetative growth stage, flowering and fruit setting stage, early fruit growth stage, fruit development stage and fruit maturity stage, respectively. W denotes soil moisture level, and K denotes K rate. Different lowercase letters indicate significant difference among treatments at the level of 0.05.
